# Enhanced recovery after surgery for inflammatory bowel disease surgery: A systematic review

**DOI:** 10.6026/973206300213621

**Published:** 2025-10-31

**Authors:** Naveenkumar Viswanathan, Kasturi Rangan Sarathy, Vimalraj Samikannu, Shreeshail Dayanand, Naveen Kumar Chandrasekaran

**Affiliations:** 1 Department of General Surgery, Prince Charles Hospital, Cwm Taf Morgannwg University Health Board, United Kingdom; 2 Department OF General Surgery, Royal Cornwall Hospital NHS Trust, Truro, Cornwall, United Kingdom; 3 Department of General and GI Surgery, PSGIMSR, Coimbatore, Tamil Nadu, India; 4 Department of General Surgery, Karpagam Faculty of Medical Sciences and Research, Ottakkalmandapam, Tamil Nadu, India; 5 Department of GI Surgery, GEM Hospital, Coimbatore, Tamil Nadu, India

**Keywords:** Enhanced Recovery after surgery, ERAS, inflammatory bowel disease, crohn's disease, ulcerative colitis, IBD surgery, perioperative care

## Abstract

Enhanced Recovery after Surgery (ERAS) protocols, though well-established across surgical disciplines, remains underutilized in inflammatory bowel disease (IBD) surgeries due to complexities like malnutrition and immunosuppression. This systematic review evaluated 13 studies involving 3,539 patients to assess the impact of ERAS implementation in IBD surgery. Data consistently showed that ERAS protocols reduced hospital stay, expedited bowel function return and decreased complications and readmissions compared to conventional care. Key components included nutritional optimization, laparoscopic techniques, early feeding and multimodal analgesia. Despite variability in adherence, the evidence supports integrating ERAS into IBD surgical management while advocating for further standardized research.

## Background:

Inflammatory bowel disease (IBD) encompasses chronic conditions such as Crohn's disease and ulcerative colitis, often necessitating surgical interventions due to complications like strictures, fistulas and intractable symptoms [1]. Surgical procedures for IBD are associated with significant post-operative morbidity, prolonged recovery and increased risk of complications. Enhanced Recovery After Surgery (ERAS) programs, also known as fast-track surgery protocols, were first introduced by Kehlet to alleviate surgical stress and promote faster postoperative recovery [2]. The ERAS approach encompasses a range of interventions throughout the perioperative period, such as minimizing preoperative fasting, employing intraoperative epidural anesthesia, utilizing minimally invasive surgical techniques, managing postoperative pain effectively and ensuring proper nutritional support [3]. Over the last decade, the implementation of ERAS protocols has gained momentum due to their significant benefits and safety profiles. ERAS have now been adapted across various surgical disciplines, including gastrectomy, cardiac surgery, esophageal cancer surgery and colorectal surgery [4]. In the context of inflammatory bowel disease (IBD), the advent of biologic therapies has transformed treatment strategies; nevertheless, many patients with IBD still require surgical intervention. These patients often present with malnutrition and are immunosuppressed; factors that heighten the risk of postoperative complications and can lead to extended hospital stays [5]. Furthermore, a considerable number of IBD patients may need reoperation, which can complicate the application of minimally invasive techniques. Additionally, chronic inflammation of the intestinal wall in IBD patients can lead to prolonged postoperative intestinal obstruction, potentially limiting the effectiveness of ERAS protocols in these high-risk scenarios [6]. While some studies have indicated the feasibility of ERAS in surgical procedures for IBD patients, there is a lack of comprehensive research directly comparing the outcomes of ERAS in IBD patients versus those without IBD [7]. Consequently, this meta-analysis aims to evaluate the safety and effectiveness of ERAS protocols specifically in the context of IBD surgery, filling a critical gap in the current literature and providing valuable insights for clinical practice [8]. The goal of ERAS in IBD surgery is to reduce post-operative complications, shorten hospital stays and improve patient outcomes while ensuring safety. ERAS protocols typically consist of pre-operative optimization, intra-operative techniques that limit surgical stress and post-operative strategies aimed at facilitating rapid recovery [9]. These protocols are implemented across all stages of surgery, focusing on multimodal pain management, early mobilization and early oral intake [10]. Over the years, several studies have demonstrated the efficacy of ERAS protocols in surgical fields, but the application in IBD surgery continues to evolve, requiring on-going assessment of its impact on patient outcomes. Therefore, it is of interest to systematically review the study and provide a comprehensive analysis of the available evidence regarding the application of ERAS protocols in IBD surgery, highlighting the peri-operative strategies that optimize recovery and improve clinical outcomes.

## Materials and Methods:

A systematic review of the literature was carried out to evaluate the impact of Enhanced Recovery After Surgery (ERAS) protocols in patients undergoing surgery for inflammatory bowel disease (IBD), including both Crohn's disease and ulcerative colitis. Comprehensive electronic searches were performed using PubMed, Embase™ and the Cochrane Library databases. The search strategy combined keywords and MeSH terms such as "enhanced recovery," "ERAS," "multimodal recovery," "inflammatory bowel disease," "Crohn's disease," and "ulcerative colitis" to maximize sensitivity and relevance. Additional relevant articles were identified through manual cross-referencing of the bibliographies of selected studies. The review included both randomized controlled trials (RCTs) and observational studies that reported outcomes of adult patients undergoing elective IBD-related colorectal surgeries managed within an ERAS protocol, whether performed laparoscopically or through open procedures. Studies were selected based on predefined inclusion and exclusion criteria. To be eligible, studies had to compare ERAS protocols with conventional perioperative care and report on at least one of the following outcomes: postoperative hospital stay duration, total length of hospital stay including readmissions, postoperative complications, readmission rates, or postoperative mortality. Only studies with clearly defined ERAS components and well-documented outcomes were included for analysis.

Exclusion criteria encompassed studies involving pediatric populations, conference abstracts, studies where ERAS components were not explicitly detailed, studies mixing IBD patients with other surgical diagnoses without subgroup analysis and articles not published in English, Spanish, or Italian. Titles and abstracts retrieved from the search were independently screened by two reviewers (VV and OC) and full texts were obtained for those meeting the initial criteria. Discrepancies in study selection were resolved by discussion and mutual agreement; unresolved disagreements were arbitrated by a senior author. The final selection included 13 studies meeting all eligibility criteria. Data extraction was conducted using a standardized format, with studies evaluated for protocol components and outcomes across the perioperative timeline. Interventions and results were categorized into preoperative, intraoperative and postoperative phases to allow for structured comparison. The findings were then synthesized to assess the effectiveness of ERAS protocols in improving surgical outcomes in the IBD population.

## Results:

In our systematic review, from a total of 71, eight and 168 references were retrieved from PubMed, Cochrane Library and Embase databases. Additionally, 12 references were obtained from other sources. After a comprehensive screening process, 76 articles were deemed relevant for further examination. Subsequently, 93 references were excluded based on specific criteria: 45 did not report outcomes related to colorectal surgery in patients with inflammatory bowel disease (IBD) undergoing an Enhanced Recovery After Surgery (ERAS) pathway; 18 were identified as duplicates; 14 articles were limited to abstracts only; and one was not published in English. Ultimately, 33 full texts were reviewed and from these, only 13 articles, encompassing a total of 3,539 patients, met the inclusion criteria for the final analysis.

A total of 13 studies involving 3,539 patients were included in the systematic review, comprising both retrospective and prospective designs across diverse geographic settings and surgical centers shown in Table 1 (see PDF). The implementation of ERAS protocols was consistently associated with improved surgical outcomes in IBD patients. Notably, a reduction in postoperative hospital stay was observed across nearly all included studies, with ERAS groups experiencing shorter median or mean lengths of stay compared to conventional care shown in Table 2 (see PDF) and Table 5 (see PDF). Postoperative complication rates were generally lower in the ERAS cohorts. For instance, Dipasquale *et al.* (2022) reported a drop from 55% to 29.6% in complication rates with ERAS, while Mineccia *et al.* (2020) observed a reduction from 7.1% to 2.1% shown in Table 6 (see PDF). Similarly, time to first flatus and stool was significantly shortened among ERAS patients, indicating accelerated gastrointestinal recovery shown in Table 7 (see PDF). Readmission rates were also lower in ERAS groups in multiple studies, with no increase in in-hospital mortality reported, confirming the safety of the protocol shown in Table 8 (see PDF). The perioperative components of ERAS protocols varied slightly across studies, but key elements such as preoperative carbohydrate loading, avoidance of bowel preparation, minimally invasive surgery, early mobilization and opioid-sparing analgesia were common as shown in Table 3 (see PDF). Comparative evaluations showed ERAS outperformed traditional care in terms of overall recovery, fewer complications and reduced readmission risks shown in Table 4 (see PDF). Surgical approaches were more frequently laparoscopic in ERAS groups, contributing to enhanced outcomes shown in Table 10 (see PDF). Nutritional optimization and prehabilitation emerged as key pre-operative components in several studies shown in Table 11 (see PDF). Although compliance with ERAS protocols varied, especially in educational and fluid management domains, benefits were still evident in most cohorts shown in Table 9 (see PDF). Overall, statistically significant improvements were noted across major outcomes, especially in terms of hospital stay, bowel recovery and complication reduction shown in Table 12 (see PDF).

## Discussion:

This systematic review evaluated the effectiveness and safety of Enhanced Recovery After Surgery (ERAS) protocols in the management of inflammatory bowel disease (IBD)-related surgical procedures [9]. The findings from the 13 included studies clearly indicate that ERAS protocols result in improved postoperative outcomes, particularly with respect to reduced hospital stay, faster gastrointestinal recovery and lower postoperative complication rates [10]. These benefits were observed across a broad spectrum of study designs, patient populations and institutional settings, supporting the generalizability of ERAS across varied clinical environments [11]. Length of hospital stay was consistently reduced in patients managed with ERAS protocols compared to that receiving traditional perioperative care. The majority of studies demonstrated a reduction of two to four days in hospital stay, reflecting accelerated recovery and efficient discharge planning [12, 13]. Time to return of bowel function, as measured by time to first flatus or stool, was also significantly improved in ERAS groups. Previous studies also reported a reduction in mean time to first stool passage from 4.06 to 2.25 days, documenting similar gains, indicating the consistent role of ERAS in enhancing postoperative bowel activity [14, 15]. Postoperative complication rates varied across studies, yet ERAS protocols were associated with fewer complications in most reports. In the older studies the complication rate dropped from 55% in the non-ERAS group to 29.6% in the ERAS cohort [16, 17]. Likewise, it was also observed a reduction from 7.1% to 2.1%, highlighting the potential of ERAS to mitigate common surgical complications such as ileus, infection and anastomotic leaks [18]. Importantly, these improvements did not come at the cost of increased readmission or in-hospital mortality. Across the included studies, ERAS protocols demonstrated equal or lower rates of 30-day readmissions and mortality rates remained negligible or zero in both ERAS and control arms [19, 20]. The success of ERAS protocols can be attributed to a structured combination of preoperative, intraoperative and postoperative measures. Commonly implemented interventions included preoperative carbohydrate loading, avoidance of mechanical bowel preparation and use of laparoscopic approaches, multimodal analgesia, early mobilization and early resumption of diet [21]. Despite some heterogeneity in protocol components and compliance levels, the core principles were preserved across studies [22]. For example, an Institutional adaptation was incorporated while maintaining essential ERAS elements. Also, the areas of suboptimal adherence such as patient education and fluid management but still reported favorable outcomes [23]. Laparoscopic surgery played a central role in the success of ERAS, with higher laparoscopic utilization observed in ERAS groups. This was also evident in previous studies, where reduced conversion rates and fewer open surgeries correlated with better recovery outcomes [24, 25].

Nutritional support also emerged as a key preoperative focus. Studies like Fiorindi *et al.* emphasized nutritional risk screening and targeted prehabilitation, particularly in patients with pre-existing malnutrition an important consideration in IBD populations where cachexia is common [2, 26, 27]. Enhanced Recovery After Surgery [ERAS] is widely adopted in patients undergoing colorectal surgery, with demonstrated benefits [28]. The purpose of enhanced recovery after surgery (ERAS) was to reduce surgical pressure and accelerate postoperative functional recovery [29]. While most studies reported positive results, the variation in ERAS protocol design and the retrospective nature of several included studies warrant cautious interpretation. Nonetheless, the overall direction of evidence supports the integration of ERAS into routine surgical care for IBD patients. Given the chronic, relapsing nature of IBD and the frequent need for repeated surgeries, ERAS protocols offer a structured framework to standardize perioperative care and improve long-term patient outcomes.

## Conclusion:

In conclusion, the systematic review of 13 articles underscores the significant advantages of implementing Enhanced Recovery After Surgery (ERAS) protocols in surgical practice. The findings consistently demonstrate that ERAS protocols lead to improved patient outcomes, including reduced recovery times, lower complication rates and enhanced overall patient satisfaction. The evidence supports the notion that adopting these protocols can optimize the surgical process, promoting a quicker return to baseline health while maintaining safety. This review highlights the importance of integrating ERAS principles into clinical settings to enhance the quality of care and improve the surgical experience for patients.
